# Antioxidant, Anti-Inflammatory and Anti-Proliferative Properties of *Stachys circinata* on HepG2 and MCF7 Cells

**DOI:** 10.3390/plants12122272

**Published:** 2023-06-11

**Authors:** Wassila Slimani, Margherita Maioli, Sara Cruciani, Sakina Zerizer, Sara Santaniello, Zahia Kabouche, Donatella Coradduzza, Mario Chessa, Silvia Fancello, Rossana Migheli, Pier Andrea Serra, Guy D’hallewin

**Affiliations:** 1 Laboratoire d’Obtention de Substances Thérapeutiques (L.O.S.T), Département de Chimie, Université des Frères Mentouri-Constantine, Constantine 25000, Algeria; 2 Department of Biomedical Sciences, University of Sassari, Viale San Pietro 43/B, 07100 Sassari, Italydonatella.coradduzza0@gmail.com (D.C.); 3 Department of Medicine, Surgery and Pharmacy, Sassari University, Viale San Pietro 43/b, 07100 Sassari, Italy; mchessa@uniss.it (M.C.);; 4 Institute of Sciences of Food Production, National Research Council, Traversa la Crucca, 3. Loc Baldinca Li Punti, 07100 Sassari, Italy

**Keywords:** natural extract, bioactive molecules, cellular mechanisms, cell proliferation, anti-proliferative activity, gene expression

## Abstract

According to the WHO, the overall age-standardized cancer rate keeps declining, and the number of cases diagnosed each year increases, remaining among the leading causes of death in 91 out of 172 recorded countries. In this context, novel cancer prediction and therapeutic protocols are compulsory. The effect of a *Stachys circinata* L’Hér dichloromethane extract (ScDME) on cell redox homeostasis and tumor proliferation was investigated. HepG2 cell feedback mechanisms to oxidative stress exposure were evaluated by determining catalase (CAT) and reduced glutathione (GSH), following the supply with ScDME (0.0–5.7 µg/µL). Cytotoxicity of ScDME against the human umbilical vein endothelial cell (HUVEC) and two human cancer cell lines (breast: MCF7; liver: HepG2) was evaluated by the MTT assay. H_2_O_2_-stressed HepG2 cells supplied with the *S. circinata* extracts exhibited significantly increased CAT and GSH activity as compared to unsupplied ones. The anti-inflammatory activity of the extracts was evaluated by real time-qPCR on IL-1, IL-6 and TNF-α expression. As a result, this research points out that *S. circinata* dichloromethane extract owns anti-inflammatory and anti-proliferative properties against MCF7 and HepG2 cells and activates CAT and GSH of the HepG2 cells’ antioxidant enzyme system.

## 1. Introduction

Cardiovascular diseases and cancer are the main causes of human mortality worldwide [1]. The malignant, metastatic, self-governing cell proliferation, termed ‘cancer or tumor’, arise in mainly all organs, but lungs are the most affected, totaling 11.6% of diagnosed cases and 18.4% of total recorded deaths in combined sexes [2]. In men, mortality is also high by liver and stomach cancer, while in woman, breast cancer is the main cause of death [3]. Noteworthy, cancer epidemiology substantially differs among and within countries, depending on age and sex, socioeconomic status and lifestyle [4,5]. Indeed, early diagnosis, access to health care and right/personalized therapies result in a significant improvement of cancer survival. Among therapies, surgery is the best option, but is limited to confined metastases (~10–15% of cases). Other cures include radiotherapy, chemotherapy, targeted therapy, virotherapy, immune checkpoint inhibitor therapy, vaccine, and combinations of them [6]. Nevertheless, tumor recurrence, drug resistance, treatment toxicity, and high heterogeneity of cancer cells indicate the need for new personalized therapies and molecules able to control a broader range of cancer cell aberrations [7,8,9]. Carcinogenesis generates a burst in intracellular reactive oxygen species (ROS) affecting survival of neighboring somatic cells and regulating tumor development [10]. Free radical overproduction causes an imbalance in the cellular redox homeostasis with oxidative damage to biomolecules (lipids, proteins, DNA) [11] and chronic inflammation, which has major implications in the etiology of chronic diseases such as cancer, diabetes, and cardiovascular conditions [12]. Therefore, it could be interesting to identify novel therapeutic drugs for cancer control that are able to affect the redox status of cancer cells [13]. Additionally, inflammation contributes to the development and progression of cancer by producing cytokines such as IL-6 and TNFα [14], which increases the release and accumulation of ROS inside of damaged tissue [15].

During metabolic processes, ROS are normally generated, and in healthy humans, the concentration of these dangerous molecules is controlled by cellular antioxidant enzymes such as superoxide dismutase (SOD), catalase (CAT), glutathione peroxidase (GPx), glutathione (GSH), glutathione reductase (GR) and antioxidant molecules [16,17]. Nevertheless, controversial issues concerning the interference between chemotherapy, ROS and antioxidants needs to be clarified to improve combined therapies [18,19,20,21]. Within this context, more and more molecules and/or phytocomplexes have been reported to influence intracellular levels of free radicals associated with carcinogenesis, some acting as antioxidants and others as antioxidant enzyme inducers [22,23]. The interest in phytotherapeutic drugs is growing, considering their potential use in combination with other approaches and/or supplements together with diet, in the recovery stage to prevent tumor recurrence [24,25,26] and drug resistance, skipping side effects generated by main therapies [27].

The genus *Stachys* (Lamiaceae), widely known in folk medicine, contains 300 species with a worldwide distribution. In Algeria, this genus is represented by 14 species [28]. The presence of flavonoids, phenylethanoid glycosides, diterpenes, saponins, terpenoids and steroids has been reported in most *Stachys* species [29]. Recently, Slimani et al. (2020) have revealed that the dichloromethane extract of *Stachys circinata* exerts immunostimulant and anti-arthritic activity on formalin-induced arthritis in mice [30]. In addition, besides the antioxidant activity, the study of Laggoune et al. (2016) has shown that the n-butanolic extract of the aerial parts of *Stachys mialhesi* exhibited a significant anti-nociceptive and anti-inflammatory activity in experimental in vivo trials [31,32]. In vivo studies in animal models on *Stachys pilifera* extracts revealed significant anti-inflammatory effects [33] and considerable cytotoxic and anti-proliferative properties on the HT-29 colorectal cell line [29]. Within this context, the present study was carried out to investigate the antioxidant and antiproliferative effects of ScDME on HepG2, MCF7 and HUVEC cells.

## 2. Results

### 2.1. Extract Characterization

The results of GC-MS analysis on the dichloromethane extract of *Stachys circinata* confirm what was previously observed by other authors [32]. In addition, terpene and phenolic compounds were observed. In particular, o-Cymene, thymol, syringol, vanillin, eugenol, syringaldehyde, hexadecanoic acid, octadecanoic acid and octadecadienoic acid were identified. All the detected compounds are observable in the Supplementary Material.

### 2.2. Effect of Stachys circinata Extracts on Cell Proliferation 

**Figure 1:**
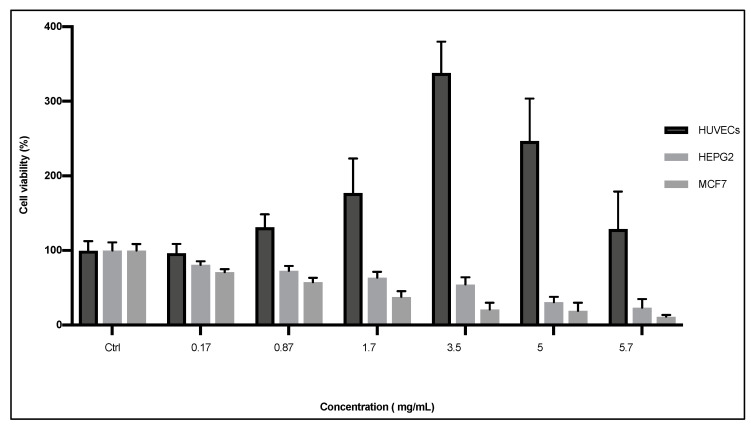
Dose-dependent cytotoxic activity of *S. circinata* extract on HUVEC, HepG2 and MCF7 cell lines. Cells were treated with increasing doses of the extract. “Ctrl” represents the cells cultured in the growing medium alone. Each cell type was incubated with the extracts for 24 h at 37 °C and subjected to MTT assays to measure % cell viability. The data were obtained from three independent assays using three wells for each assay.

Cytotoxic effects of ScDME were quantified by the MTT assay (Figure 1). *S. circinata* dichloromethane extract caused a high decrease in both HepG2 and MCF7 cells viability (*p* = 0.000), with values ranging from 23.48 to 80.72% and from 11.23 to 70.94% 24 h after the treatments, respectively. Moreover, *S. circinata* extract inhibited MCF7 and HepG2 cell growth in a dose-dependent manner. The most significant cytotoxicity effect was achieved in cells exposed to the higher concentrations of ScDME. The extract showed selective cancer cell line cytotoxicity with IC50 values of 3.67 and 4.87 mg/mL against breast cancer cells (MCF7) and liver cancer cells (HepG2), respectively, whereas ScDME showed no cytotoxic effect on normal cells (HUVEC) with values ranging from 96.44 to 337.8%. The extract promoted HUVEC cell proliferation at the concentrations ranging from 0.17 to 5.7 mg/mL, and high cell viability at the concentration of 3.5 mg/mL of the extract.

### 2.3. Effect of Stachys circinata Extracts on Catalase Activity and Glutathione Concentrations

**Figure 2:**
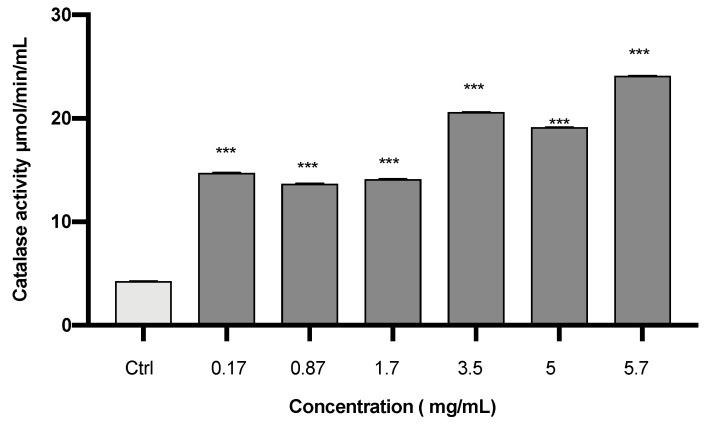
Effect of *S. circinata* extracts on cellular catalase activity. Data are expressed as mean ± standard error of the mean (SEM) (n = 3; *** *p* ≤ 0.001).

Our data showed that the CAT activity was highly significant in cells treated with different concentrations of *S. circinata* extract as compared to the control group (*p* = 0.000) (Figure 2).

**Figure 3:**
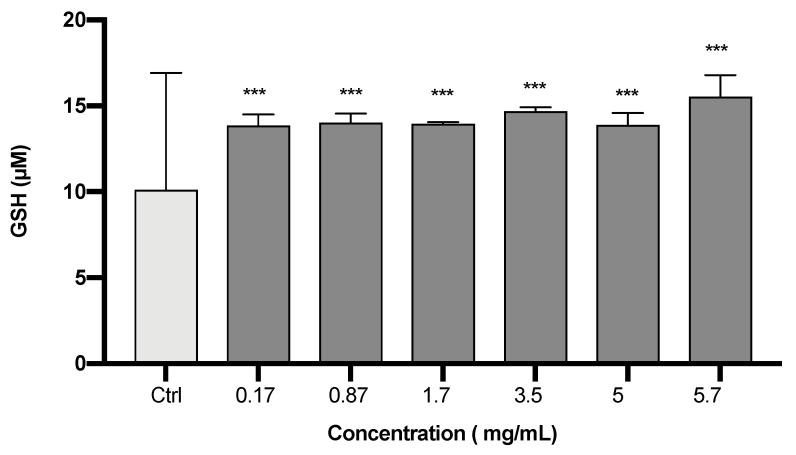
Effect of *S. circinata* extracts on cellular GSH levels. Data are expressed as mean ± SEM (n = 3; *** *p* ≤ 0.001).

Figure 3 shows the effect of *S. circinata* extract on the levels of GSH, which were significantly enhanced in treated cells as compared to the control group (*p* = 0.000).

### 2.4. Effect of Stachys circinata Extracts on ROS Concentrations

**Figure 4:**
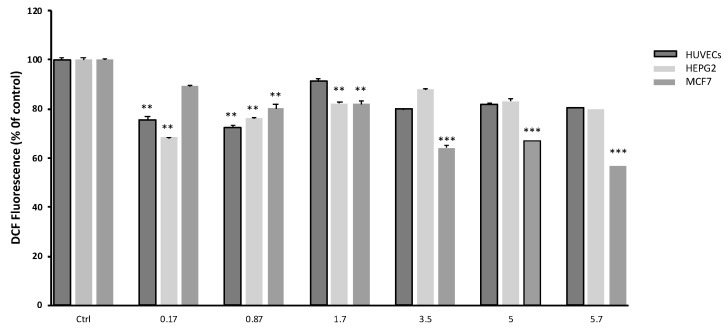
Effect of *S. circinata* extracts on cellular ROS levels on HUVEC, HepG2 and MCF7 cell lines. Data are expressed as mean ± SEM (n = 3; ** *p* ≤ 0.01, *** *p* ≤ 0.001).

Figure 4 shows the levels of reactive oxygen species (ROS) on HUVEC, HepG2 and MCF7 cells. ROS levels were significantly decreased in treated cells, as compared to controls, confirming the increased activity of catalase and glutathione detected in treated cells. 

### 2.5. Effect of Stachys circinata Extract on the Expression of Pro-Inflammatory Cytokines

**Figure 5:**
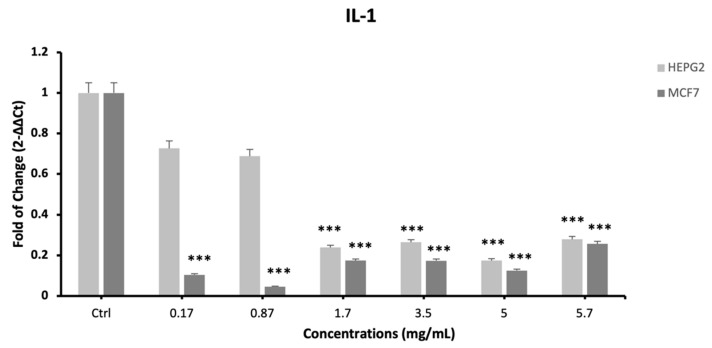
Effect of *S. circinata* extracts on IL-1 expression levels. The mRNA levels for each gene were normalized to β-Actin and expressed as fold of change (2^−∆∆Ct^) of the mRNA levels observed in undifferentiated control cells defined as 1 (mean ± SD; n = 6). Data are expressed as mean ± SD referred to the control (*** *p* ≤ 0.001).

**Figure 6:**
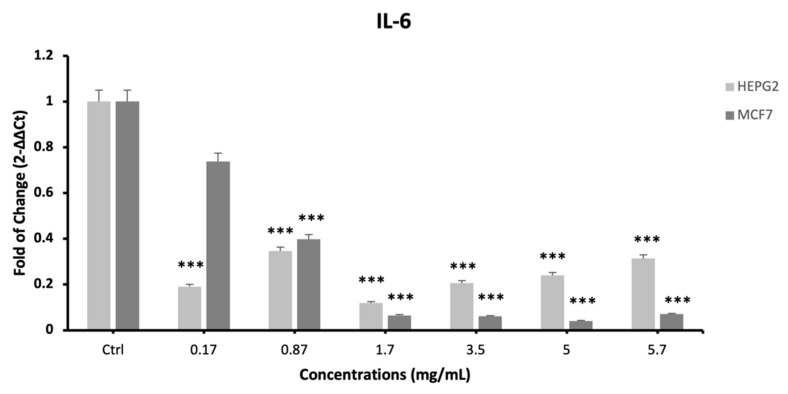
Effect of *S. circinata* extracts on IL-6 expression levels. The mRNA levels for each gene were normalized to β-Actin and expressed as fold of change (2^−∆∆Ct^) of the mRNA levels observed in undifferentiated control cells defined as 1 (mean ± SD; n = 6). Data are expressed as mean ± SD referred to the control (*** *p* ≤ 0.001).

**Figure 7:**
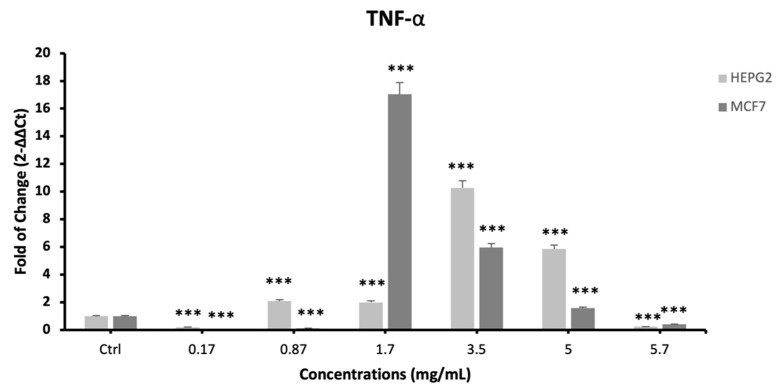
Effect of *S. circinata* extracts on TNF-α expression levels. The mRNA levels for each gene were normalized to β-Actin and expressed as fold of change (2^−∆∆Ct^) of the mRNA levels observed in undifferentiated control cells defined as 1 (mean ± SD; n = 6). Data are expressed as mean ± SD referred to the control (*** *p* ≤ 0.001).

The expression of proinflammatory cytokines IL-1, IL-6 and TNF-α was evaluated by qPCR (Figure 5; Figure 6 and Figure 7, respectively) in cells exposed to different concentrations of the extract. The mRNA levels of IL-1 and IL-6 significantly decreased after 24 h of treatment (Figure 5 and Figure 6), as compared to control untreated cells, for all the tested concentrations.

At the same time, TNF-α showed a completely different trend, with a significant downregulation when cells were exposed to 0.17, 0.87 and 5.7 mg/mL of the extracts (Figure 6), and a significant upregulation for the intermediate concentrations (0.17 mg/mL), and then decreasing with higher concentrations, as compared to control untreated cells. 

## 3. Discussion

Several degenerative diseases related to aging, including cancer, cardiovascular disease, cataracts and diabetes, are the consequence of oxidative stress damage induced by free radicals. These chemical species carrying one or more unpaired electrons are highly reactive and unstable. Interestingly, antioxidants deriving from natural compounds in medicinal plants, fruit and vegetables can trap and neutralize free radicals [34]. 

Within this context, we evaluated the ability of ScDME to counteract the proliferation of HepG2 and MCF7 cells, without affecting the viability of HUVEC cells. Ferhi et al. (2019) revealed that the extracts obtained from grape leaves grown in the Medea region (Algeria) exhibited an antiproliferative effect on MCF7 and HepG2 cells, without inducing damage to HUVEC as a non-cancerous cell [35]. Furthermore, Jassbi et al. (2014) reported that due to the presence of cytotoxic compounds with different polarities in several *Stachys* species such as *S. pilifera*, dichloromethane extract (that may hold more nonpolar agents such as terpenoids) showed a stronger antiproliferative effect [36]. Nevertheless, some plants such as *S. persica*, 80% methanol extract (containing more polar molecules as phenolic compounds), have even exhibited higher cytotoxic effects. A previous study also demonstrated that the chloroform fraction of *S. setifera* greatly inhibited the proliferation of the breast ductal carcinoma cell line (T-47D) (IC50 2.44 μg/mL), as compared to normal cells (IC50 394.88 μg/mL) whose major components are terpenoids and flavonoids [37]. Seelinger et al. (2008) reported that luteolin, isolated from two Asian plants traditionally used as anticancer medicines, *Epimedium koreaonum* and *Terminalia arjuna*, was able to inhibit MCF7 and HepG2 proliferation in a dose-dependent manner [38]. The cytotoxic effect of luteolin and apigenin was also previously demonstrated on human chronic myelogenous leukemia (K562) and bladder cancer (RT112) cells, in a dose- and time-dependent manner [39], with increased ROS generation [40]. Furthermore, apigenin also exhibited broad anticancer effects in various human cancers. This flavone inhibits cancer cell proliferation by triggering cell apoptosis, inducing autophagy and modulating the cell cycle [41]. In other studies, isorhamnetin was able to inhibit lung cancer cell proliferation in vitro and in vivo, and was also able to counteract other carcinoma cell line proliferation, such as MCF7. Its mechanisms of action may involve cell apoptosis by down-regulating oncogenes and inducing apoptotic genes [42]. In another study, oleanolic acid exerted cytotoxic activity against HepG2 by arresting the cell cycle and inducing apoptosis and DNA fragmentation [43]. Interestingly, Zarei and Yaghoobi (2017) reported that the extracts of *Fritillaria imperialis* L. were toxic for human liver cancer cells (LCL-PI 11) and breast adenocarcinoma cells (MCF7), probably inducing cell cycle arrest or intrinsic apoptosis [44]. These results are further inferred by the work of Aghbali et al. (2013), reporting that *Vitis vinifera* had a pro-apoptotic effect, inhibiting cell growth, whereas no cytotoxic activity was observed on HUVEC cells [45]. 

From the dichloromethane extract of *S. circinata*, a variety of secondary metabolites were isolated, and fifteen known compounds have been identified. Among them, flavonoids such as luteolin, apigenin, isorhamnetin; triterpenoids such as betulinic acid, ursolic acid and olealonic acid; and sterols such as stigmasterol and β-sitosterol [32]. Here, we demonstrated that *S. circinata* extract led to a selective cytotoxic activity against MCF7 and HepG2 cell lines, while no cytotoxicity against HUVEC could be observed. This effect is probably related to the high amount of flavonoids detected in the dichloromethane extract.

To investigate the effect of *S. circinata* extract on HepG2 cells, the activity levels of CAT and GSH were measured. The potent antioxidant properties of flavonoids are well established, disclosing their ability to regulate the enzymes involved in oxidative stress processes, such as SOD, CAT and GR [46]. This antioxidant capability is due to the presence of hydroxyl groups (·OH) in the skeleton of these classes of molecules. Moreover, other authors have proven that phenolic and flavonoid contents are associated with antioxidant properties, rendering them excellent stabilizers for lipid peroxidation [47]. 

Reduced glutathione is a natural antioxidant produced inside the cell, playing both a role of co-factor for glutathione peroxidase and an active scavenger to eliminate reactive species, such as hydroxyl radicals, carbon-centered radicals, peroxynitrite and a singlet oxygen molecule. The role of GSH is to preserve cellular redox status. It is possible that an increase in GSH levels will minimize ROS levels, thus antagonizing oxidative stress [48]. Catalase is present in all aerobic cells, but the highest concentration is found in the liver and erythrocytes [49]. 

The CAT and GSH activities of cells treated with ScDME were significantly higher than the control. However, the triggering of CAT and GSH activities observed in cells treated with ScDME infer the antioxidant activity of the extract. Moreover, it is largely demonstrated that ROS production is strictly related to cancer progression. Inside of the tumor microenvironment, there are inflammatory cells that contribute to ROS production [50]. Cancer cells are found within a tumor microenvironment (TME) in which other cell types, including inflammatory immune cells, also coexist [51]. The chronic inflammation resulting from the secretion of pro-inflammatory cytokines can promote cancer progression by directly shaping the TME [14]. For this reason, we evaluated the expression of IL-1, IL-6 and TNF-α by real-time-qPCR. ScDME showed a significant ability in modulating IL-1 and IL-6 expression levels, which decreased in treated cells, as compared to untreated controls. TNF- α showed a completely different trend. As already demonstrated by other authors, TNF-α induces autophagy through the ERK1/2 pathway, reducing their proliferation and clonogenic capability [52,53]. Our results show that cells treated with 0.17 and 3.50 mg/mL of the ScDME exhibited increased TNF- α expression, as compared to untreated controls. This increased expression would confirm the ability of the extracts to block proliferation by influencing cell behavior. In conclusion, *S. circinata* could be a valid aid against tumor cell proliferation in the future by inducing antioxidant enzymes, lowering cancer cell viability and modulating pro-inflammatory cytokines involved in tumor progression. 

## 4. Materials and Methods

### 4.1. Reagents and Cells

Dulbecco’s phosphate-buffered saline (DPBS) (Euroclone, Milano, Italy); Dulbecco’s Modified Eagle’s Medium (DMEM) (LONZA, BioWhittaker^®,^ Morrisville, NC, USA); Minimum Essential Medium and Non-Essential Amino Acids (MEM, NEAA) (Gibco^®^, Life Technologies™. Grand Island, NY, USA); endothelial cell basal medium (EBM) (LONZA, Clonetics^®,^ Morrisville, NC, USA); fetal bovine serum (FBS) (Life Technologies. Grand Island, NY, USA); L-glutamine, penicillin, streptomycin. Bovine brain extract (BBE), epidermal growth factor (rhEGF), gentamicin sulfate, ascorbic acid, and hydrocortisone (Euroclone, Milano, Italy); *(3-(4,5-dimethylthiazol-2-yl)-2,5-diphenyltetrazolium bromide*) tetrazolium reduction (MTT), cell proliferation assay ATCC^®^ 30-1010K kit (Invitrogen Co). Dimethyl sulfoxide (DMSO) (Sigma Aldrich, St. Louis, MO, USA, ).

The human cancer cells HepG2 (hepatocarcinoma cell line) were obtained from ATCC (American Cell Culture Collection), MCF7 (breast cancer cell line) from the pathological anatomy (Civil hospital of Cagliari) and normal human umbilical vein endothelial cell (HUVEC CC-2519) from LONZA (Clonetics^®,^ Morrisville, NC, USA).

### 4.2. Plant Collection and Authentication

Aerial parts of *S. circinata* L’Her were collected from Djebel El-Ouahch-Constantine (Northeastern Algeria) in April 2013 during the flowering stage. A voucher specimen (LOST SC04/13) has been deposited in the Laboratory of therapeutic substances, University frères Mentouri-Constantine and authenticated by Prof. G. De Belair (University of Annaba, Algeria).

### 4.3. Preparation of the Dichloromethane Extract

Air-dried and powdered aerial parts (1 kg) of *S. circinata* were macerated three times at room temperature (25C) with MeOH-H_2_O (7:3, *v*/*v*) for 24 h. After filtration, the filtrate was concentrated and dissolved in water (600 mL) to obtain the aqueous phase. The resulting solution was extracted successively with a liquid-liquid extraction method by mixing the aqueous phase with a range of solvents with decreased polarity in the following order: petroleum ether (PET), dichloromethane (CH_2_Cl_2_), ethyl acetate (EtOAc) and finally with n-butanol (n-BuOH). Then, all extracts were evaporated using a rotavapor and vacuum stored at −20 °C. In all experiments, the recovered CH_2_Cl_2_ extract was employed in all subsequent experiments. 

Concentration in a vacuum at room temperature led to the following extract recoveries: PET (2.3 g); CH_2_Cl_2_ (9 g); EtOAc (5 g) and n-BuOH (25 g). The resulting dichloromethane extract of *S. circinata* (ScDME) was then used in all experiments.

### 4.4. GC-MS Set-Up

Gas chromatography–mass spectrometry (GC-MS) analysis was carried out using a Hewlett Packard 5890 GC-MS system operating in EI mode at 70 eV and equipped with a HP-5 capillary column (30 m × 0.25 mm, film thickness 0.25 mm). The column temperature was held at 50 °C for 2 min, then increased to 300 °C at a rate of 5 °C/min and held at 300 °C for 10 min. Injector and detector temperatures were 300 °C. 

Helium was used as the carrier gas at a flow rate of 1 mL per minute and a split ratio of 1:10.

### 4.5. Cell Culture 

MCF7 and HepG2 were kept in Dulbecco’s Modified Eagle Medium (DMEM) amended with (*v*/*v*) 10% of FBS, 1% of antibiotic P/S (penicillin-streptomycin), 1% of glutamine and 1% of MEM and NEAA. HUVEC cells were cultured in EBM containing 2% FBS, 1% P/S, 0.4% BBE, 0.1% of EGF, 0.1% gentamicin sulfate, 0.1% ascorbic acid, and 0.1% hydrocortisone. Cells were grown in 75-cm^2^ tissue culture flasks in a culture incubator at 37 °C with 5%CO_2_ and saturated humidity.

### 4.6. MTT Viability Assay

The antiproliferative activity of the *S. circinata*-CH_2_Cl_2_ extract on HepG2, MCF7 and HUVEC cells was determined using the MTT reduction assay. This rapid colorimetric method is based on the OD shift following the yellow tetrazolium salt cleavage to the purple formazan crystals by the succinate dehydrogenase of functional mitochondria (Mosmann 1983). In short, once HepG2, MCF7 and HUVEC cells reached the exponential growth phase, cells were seeded on a 96-well plate at a concentration of 5,000 cells/well in 100 µL of medium and incubated at 37 °C in a 5% CO_2_ atmosphere incubator (Steril-Cycle CO_2_ Incubator HEPA Class100, Thermo Electron Corporation, Waltham, Massachusetts, USA). After 24 h incubation, the medium was replaced with a fresh one containing 0.17, 0.87, 1.7, 3.5, 5 and 5.7 mg/mL of the ScDME, and cultures were returned at 37 °C with 5% CO_2_ for an additional 24 h. For the negative control, the medium was only refreshed (0.0 mg/mL of ScDME). Finally, the medium was removed and substituted with 100 μL of MTT prepared in sterile DPBS (0.65 mg/mL), and then, cells in culture were incubated for an additional 2 h at 37 °C with 5% CO_2_ and saturated humidity. After incubation, the MTT-DPBS was removed and replaced by 200 µL/well of DMSO to solubilize the formazan crystals. The solubilized purple formazan was quantified with a spectrophotometer at 578 nm with background subtraction at 630–690 nm (Gemini EMMicroplate Reader-Molecular devices). Experiments were performed in triplicate. Optical densities were used to determine the % of cell proliferation using the formula:

% Cell proliferation = (At − Ab⁄Ac − Ab) × 100

where,

At = Absorbance value of test compound (ScDME)

Ab = Absorbance value of blank (Medium alone)

Ac = Absorbance value of control (Medium + Cells).

### 4.7. Antioxidant Activity

To evaluate the antioxidant activity of ScDME, glutathione and catalase were investigated in HepG2 cells exposed to the extract at a concentration of 0.17, 0.87, 1.7, 3.5, 5 and 5.7 mg/mL for 24 h.

#### 4.7.1. Glutathione (GSH) Activity

HepG2 cells were seeded in Petri dishes (5000,000 cells/6 mL per dish) and incubated for 24 h at 37 °C. GSH was quantified using a Glutathione Assay Kit (Sigma-Aldrich^®^ CS0260) according to the manufacturer’s instructions. The absorbance was measured at 405 nm and read at 1 min intervals for 5 min in a Gemini EMMicroplate Reader (Molecular devices).

#### 4.7.2. Catalase (CAT) Activity

Catalase was measured using a Catalase Assay Kit (Sigma-Aldrich^®^ CAT100). The protocol employed, according to the manufacturer’s instructions, is based on the measurement of the hydrogen peroxide produced following catalase activity. The absorbance was read at 490 nm after 15 min at room temperature for color development [Thermo Fisher Scientific, Waltham, Massachusetts, USA; Gemini EMMicroplate Reader (Molecular devices)].

#### 4.7.3. ROS Detection 

Reactive oxygen species (ROS) production was detected in normal and tumoral cells using reactive oxygen species detection reagents (Life Technologies, Grand Island, NY, USA). Cells were incubated 1 h in PBS containing 10 µM of the probe. The loading buffer was removed and replaced with growth medium. ROS concentration was detected by a spectrophotometric microplate reader using an excitation and emission wavelength at 504 nm and 529 nm, respectively.

### 4.8. Gene Expression Analysis

Total RNA was isolated from HepG2 and MCF7 cells after 24 h of treatment with different concentrations of the extracts using TRIzol reagent and quantified by Nanodrop, measuring the absorbance at 260/280 nm (NanoDrop 2000, spectrophotometer ND8008, Thermo Fisher Scientific, Waltham, MA, USA). Approximately 1 µg of total RNA was reverse transcribed to cDNA by SuperScript^®^ VILO™ cDNA Synthesis Kit (Life Technologies, Grand Island, NY, USA). The quantitative polymerase chain reaction was run in triplicate using a CFX Thermal Cycler (Bio-Rad, Hercules, CA, USA), and 2 µL of cDNA was amplified in 25 µL reactions using a Platinum Quantitative PCR Supermix UDG Kit. A Supermix 2X was mixed with Sybr Green I, 0.1 µM of primer and 10 nM fluorescein (Life Technologies, Grand Island, NY, USA). Relative target Ct (the threshold cycle) values of IL-1; IL-6 ant TNF-α were normalized to β-Actin, used as a housekeeping gene. All primers used (Thermo Fisher Scientific, Grand Island, NY, USA) are described in Table 1. The mRNA levels of cells treated with the extracts were expressed using the 2^−ΔΔCt^ method, relative to the mRNA level of the untreated sample for each experiment. 

### 4.9. Statistical Analysis

Results are presented as mean ± SEM (standard error of the mean). Statistical analyses of the data were performed using one–way ANOVA tests and Tukey’s multiple comparison tests (SPSS version 20). The values of *** *p* ≤ 0.001, ** *p* ≤ 0.01 and * *p* ≤ 0.05 were considered to indicate significant differences.

## Figures and Tables

**Table 1:** Primer sequences.

Primer Name	Forward	Reverse
β-Actin	GAGTCAACGGAATTTGGTCGT	GACAAGCTTCCCGTTCTCAG
IL-1	GCTACGAATCTCCGACCACC	ATCGTGCACATAAGCCTCGT
IL-6	TCTCAACCCCCAATAA	GCCGTCGAGGATGTA
TNF-α	CCTCAGACGCCACAT	GAGGGCTGATTAGAGAGA

## Data Availability

Data are contained within the article.

## Supplementary Materials

The following supporting information can be downloaded at: https://www.mdpi.com/article/10.3390/plants12122272/s1, Figure S1. Chemical GC- MS profile of ScDME; Table S1. GC-MS identification of compounds in ScDME.
