# Supplementary material for: Antioxidant, Anti-Inflammatory and Anti-Proliferative Properties of Stachys circinata on HepG2 and MCF7 Cells
Source: Plants (Basel). 2023 Jun 11;12(12):2272. doi: 10.3390/plants12122272 (PMC10304351; doi:10.3390/plants12122272)
Supplement: Supplementary file 1 [file plants-12-02272-s001.zip › plants-2206725-supplementary.pdf]

Supplementary material

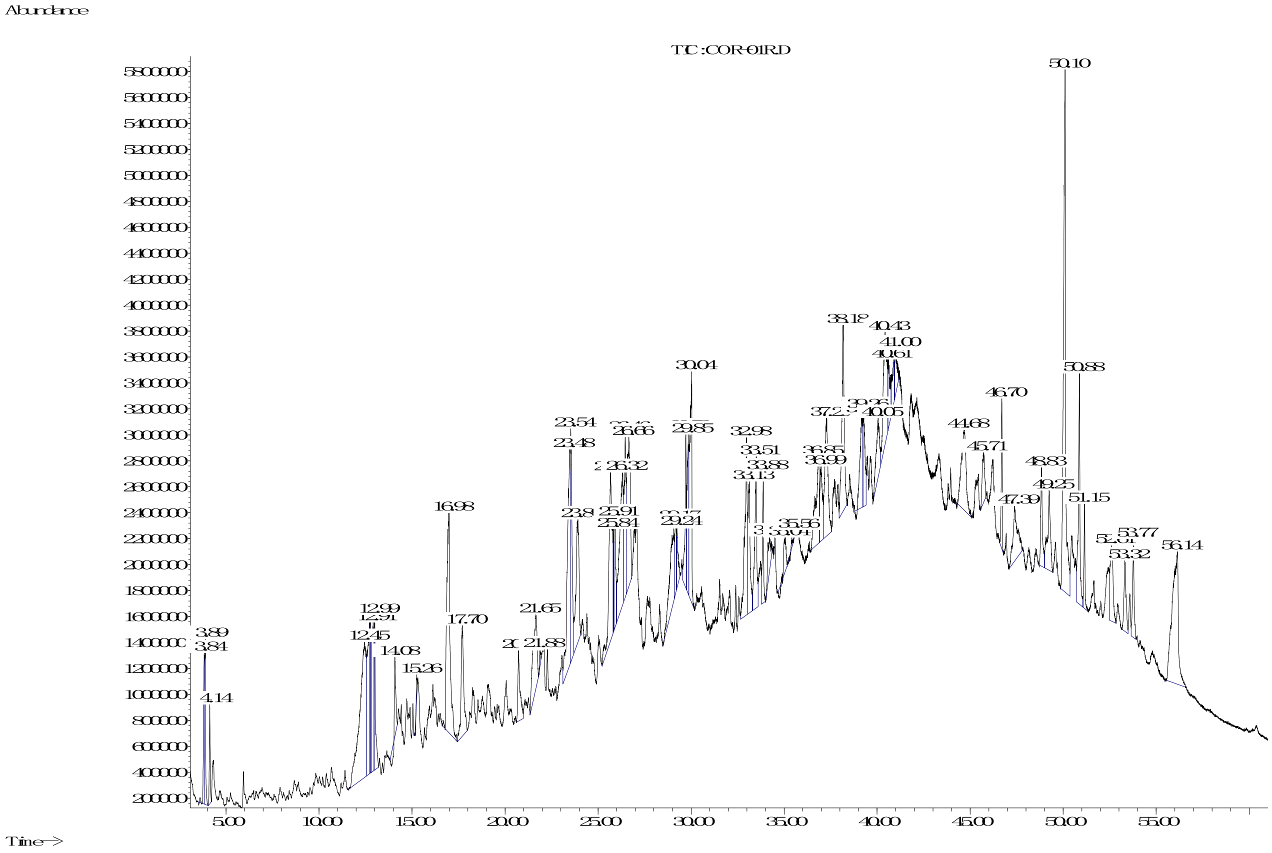

Figure 1. Chemical GC- MS profile of ScDME.

Table 1. GC-MS identification of compounds in ScDME.

	tR (min)	Compound
1	3.83	Methanamine, N,N-dimethyl
		Silane, ethenyltrimethyl
		Tetrahydrofuran, 2,2-dimethyl
2	3.89	2,3-Dimethyl-undec-1-en-3-ol
		2-Hydroxy-5,5-dimethyl-hex-2-en-4-one
3	4.13	Pentanal, 2,2-dimethyl
4	12.45	Octanal, 7-hydroxy-3,7-dimethyl
		Hexanamide
		2-Pentanone, 5-methoxy
5	12.72	Hydrazine, 1,1-bis(1-methylethyl)
		2-Hexanol, 2-methyl
6	12.77	Ethanol, pentamethyl
7	12.91	2-Heptanol, 2-methyl
		4-Hydroxy-3-hexanone
8	12.99	1-Butanol, 3-methoxy
9	14.08	2-Methoxy-4-vinylphenol
		Phenol, 2,3,5,6-tetramethyl
		Ethanone, 1-(3-methoxyphenyl)
10	15.26	Phenol, 2,6-dimethoxy

11	16.98	Vanillin
		Benzaldehyde, 3-hydroxy-4-methoxy
12	17.70	Phenol, 2-methoxy-4-(1-propenyl)
13	20.73	4-Methyl-2,5-dimethoxybenzaldehyde
		1,3,5,7-Cyclooctatetraene
		2,4,7(1H,3H,8H)-Pteridinetrione
14	21.65	1H-Imidazole, 4-methyl-5-nitro
		Pyridine-2-thiol-1-oxide
		3,5-Heptanedione, 2,2,6,6-tetra
15	21.88	Ethanone, 1-(2-hydroxy-5-methox)
		1,3,6,10-Dodecatetraene, 3,7,11
		1H-Benzocyclohepten-7-ol, 2,3,4
16	23.48	Benzaldehyde, 4-hydroxy-3,5-dim
		3,4-Dimethoxy-5-hydroxybenzalde
17	23.89	Acetaldehyde, (phenylthiol)
		4-(3-Hydroxy-2,2,6-trimethyl-7
		N-Isobutyl-(2E,4Z)-octadienamide
18	25.67	2'-Methoxy-[1,1'-biphenyl]-2-ca
		Harmine
19	25.84	1-(1-Hydroxybutyl)-2,5-dimethox
		2,4,6(1H,3H,5H)-Pyrimidinetrion
		Benzeneacetic acid, alpha-hyd
20	25.91	Benzeneacetaldehyde, alpha-ph
		9-Ethoxy-10-oxatricyclo
		1,2,4-Cyclopentanetrione
21	26.32	2(4H)-Benzofuranone, 5,6,7,7a-tetrahydro-4,4,7a-trimethyl
		1(2H)-Naphthalenone, octahydro
22	26.46	9-Borabicyclo[3.3.1]nonane
		9-Undecenal, 2,10-dimethyl
23	26.65	Hepta-2,4-dienoic acid, methyl
24	29.08	Benzenemethanol, 3,4,5-trimethoxy
		3-Hydroxy-4,5-dimethoxybenzoic
25	29.17	1,2,3,4-Tetramethoxybenzene
26	29.24	Benzoic acid, 4-hydroxy-3,5-dim
27	29.72	Hexadecanoic acid, ethyl ester
		Nonadecanoic acid, ethyl ester
28	29.85	n-Hexadecanoic acid
29	32.98	Z,Z-10,12-Hexadecadien-1-ol acetate
		7-Pentadecyne
		9,12-Octadecadienoic acid (Z,Z)
30	33.13	Cis-8-methyl-exo-tricyclo[5.2.1.0(2.6)]decane
		7-Heptadecyne, 17-chloro
		11-Dodecyn-1-ol acetate
31	33.51	Octadecanoic acid
32	33.88	1H-Naphtho[2,1-b]pyran, 4a,5,6
		1-[1-Methoxy-3,3-dimethyl-2-(3)
		Spiro[5.6]dodecane-1,7-dione
33	34.22	(1S,5R,10S)-1,5,8,8-Tetramethyl
		Ledol
34	35.04	4,8-Methanoazulen-9-ol, decahyd
		2(1H)-Naphthalenone, octahydro
		4-(1,3,3-Trimethyl-bicyclo[4.1] hept-2-yl)-but-3-en-2-one
35	35.56	1,4-Methanoazulen-7-ol, decahyd
		Methyl 2-octylcyclopropene-1-he

		4-(3,3-Dimethyl-but-1-ynyl)-4-hhydroxy-2,6,6 trimethylcyclohex-2-enone
36	36.85	Spiro[4.5]decan-7-one, 1,8-dimethyl-4-(1-methylethyl)
		Dodecane, 1,12-dibromo
		1H-1,3a-Ethanopentalen-4-ol
37	36.98	Longipinane
		1-Cyclohexene-1-carboxaldehyde
38	37.29	8-Methyl-9,11-tetradecadien-1
		1-Naphthalenemethanol, 1,4,4a,5
		(E)-3(10)-Caren-2-ol
39	38.18	4,7-Methanobenzofuran, 2,2'-oxy
		Cyclopentanecarboxylic acid
		2-Methyl-3-propenylcyclopropane
40	39.21	1,4-Methanoazulen-7(1H)-one
		2,10-Dodecadien-1-ol, 3,7,11-trimethyl
41	39.26	Cyclohexane-1-methanol, 3,3-dimethyl
		2-Dodecen-1-yl(-)succinic anhydride
		Bicyclo[2.2.1]heptane-1-methane
42	40.05	(7,7-Dimethyl-2-oxobicyclo[2.2]
		Naphtho[1,2-b]furan-2,8(3H)-dione
		Cedrol
43	40.43	Bicyclo[3.3.0]oct-2-ene
		Bicyclo[3.1.1]hept-3-en-2-ol
44	40.61	(1S,5R,10S)-1,5,8,8-Tetramethyl
		2,3,7-Trimethyl-3-vinyl-oct-6-enoic acid
45	40.91	Naphthalene, 1-[1-(bromomethyl)
		2-Dodecen-1-yl(-)succinic anhydride
		11H-Cyclopenta[a]phenanthren
46	41.01	(1,5,5,8-Tetramethyl-bicyclo[4]
		Undeca-3,4-diene-2,10-dione
		Malononitrile, 1-(1,7,7-trimethyl
47	44.68	(E)-2-Caren-4-ol
		8a(2H)-Phenanthrenol, 7-ethenyl
48	45.71	Benzaldehyde, 2-hydroxy
		Estra-1,3,5(10)-triene-3,17-diol
		Phenanthro[3,2-b]furan-4-methan
49	46.71	Rishitin
		Stigmastan-6,22-dien, 3,5-dedihydro
		1,1-Dimethyl-1-phenyl-4,4,4-trifluoro
50	47.39	Butyl pentafluorobenzyl ethylph
		Propanedioic acid, methyl[(3,4)
		6-(1-Hydroxy-ethyl)-2-iodo-4-ox
51	48.83	2-Pentenoic acid, 5-(decahydro-)
		2-Pyrrolidinecarboxamide
		5 alpha-Pregnan-20-one, 3 beta
52	49.25	Cholesta-5,22-dien-3-ol, (3bet)
		Pregn-5-en-20-one, 3,17-dihydro
		Benzene, 1,4-dichloro-2-nitro
53	50.10	2-Pentanone, 4-methyl-1-(triphe)
		Stigmastan-3,5-dien
		2'-Hydroxy-5-methyldihydro
54	50.88	Caryophyllene oxide
		Longifolenaldehyde
		1-Naphthalenepropanol

55	51.15	2-Methyl-5-methoxytryptamine
		Isobutenal methylphenylhydrazone
		8-Quinolinol, 2-(aminomethyl)
56	52.61	Cholan-24-oic acid, 3,12-dioxo
		(4H)1,3,2-Dioxaborin, 4-ethenyl
57	53.32	Cholestane, 4,5-epoxy-, (4 alpha-cholestane)
		2,4,4-Trimethyl-3b-hydroxymethyl
		4,7-Methano-1H-indene, octahydrydro
58	53.77	Antiquorin
		Pregn-7-en-3-ol, 20-methyl
		2,3-Diphenyl-5-methoxybenzo-1,4
59	56.14	1,2,3,3a,4,5,6,10b-Octahydrofluoren
		2,6-Pyridinedimethanol
		1,2,3-Triazol, 2-(E-4,4-dicyano)
